# A cell based assay for evaluating binding and uptake of an antibody using hepatic nonparenchymal cells

**DOI:** 10.1038/s41598-021-87912-6

**Published:** 2021-04-16

**Authors:** Yuki Noguchi, Kazuhisa Ozeki, Hiroaki Takesue, Hidetaka Akita

**Affiliations:** 1 Research Division, Chugai Pharmaceutical Co., Ltd., 1-135, Komakado, Gotemba, Shizuoka 412-8513 Japan; 2 Laboratory of DDS Design and Drug Disposition, Graduate School of Pharmaceutical Sciences, Chiba University, 1-8-1 Inohana, Chuo-ku, Chiba, Chiba 260-0856 Japan

**Keywords:** Drug discovery, Pharmaceutics

## Abstract

Evaluation of the binding and uptake of an antibody in liver non-parenchymal cells (NPC), including liver sinusoidal endothelial cells, is important for revealing its pharmacokinetic (PK) behavior, since NPC has important roles in eliminating an antibody from the blood via the Fc fragment of IgG receptor IIB (FcγRIIB). However, there is currently no in vitro quantitative assay using NPC. This study reports on the development of a cell-based assay for evaluating the binding and uptake of such an antibody using liver NPC of mice and monkeys. In mice, the FcγRIIB-expressing cells were identified in the CD146-positive and CD45-negative fraction by flow cytometry. A titration assay was performed to determine the PK parameters, and the obtained parameter was comparable to that determined by the fitting of the in vivo PK. This approach was also extended to NPC from monkeys. The concentration-dependent binding and uptake was measured to determine the PK parameters using monkey NPC, the FcγRIIB-expressing fraction of which was identified by CD31 and CD45. The findings presented herein demonstrate that the in vitro liver NPC assay using flow cytometry is a useful tool to determine the binding and uptake of biologics and to predict the PK.

## Introduction

The in vivo fitting of the PK profile is the gold standard in antibody PK assays, since it permits the PK parameters to be determined separately for non-specific and target-dependent elimination. These parameters are generally used to determine the human PK of an antibody^1,2^. However, monitoring plasma concentrations typically requires a long period of time (generally several days to months), and a large number of experimental animals, especially monkeys, are needed to evaluate the PK of an antibody in vivo. This is a problem in terms of the 3Rs (replacement, reduction and refinement of experimental animals) principle in the early stage of drug discovery. Thus, an in vitro assay which is capable of evaluating both binding and uptake via the neonatal Fc receptor (FcRn) and the Fc fragment of the IgG receptor (FcγR) IIB, which play important roles in antibody clearance would be highly desirable^3^.

Approximately 40% of hepatic cells are non-parenchymal cells (NPC), and are a mixture of various cells including liver sinusoidal endothelial cells (LSEC), kupffer cells (KC), stellate cells, among others^4^. NPC, in which various scavenger receptors are expressed, play an important role in the elimination of an antibody, an antigen–antibody complex (immune complex)^5,6^, an oligonucleotide^7,8^, a proteglycan^9,10^, and a virus^11^ from the blood circulation. LSEC and KC, which express FcγRIIB in NPC, play a key role in the clearance of an antibody and an immune complex particularly^5,6^. Thus, the binding of the Fc region of an antibody against FcγRIIB has a large influence on antibody clearance^12^. Therefore, evaluating the binding and uptake of an antibody in FcγRIIB-expressing cells in NPC, and determining the PK parameters related to FcγRIIB-mediated elimination is essential in terms of understanding the PK of an antibody.

**Figure 1:**
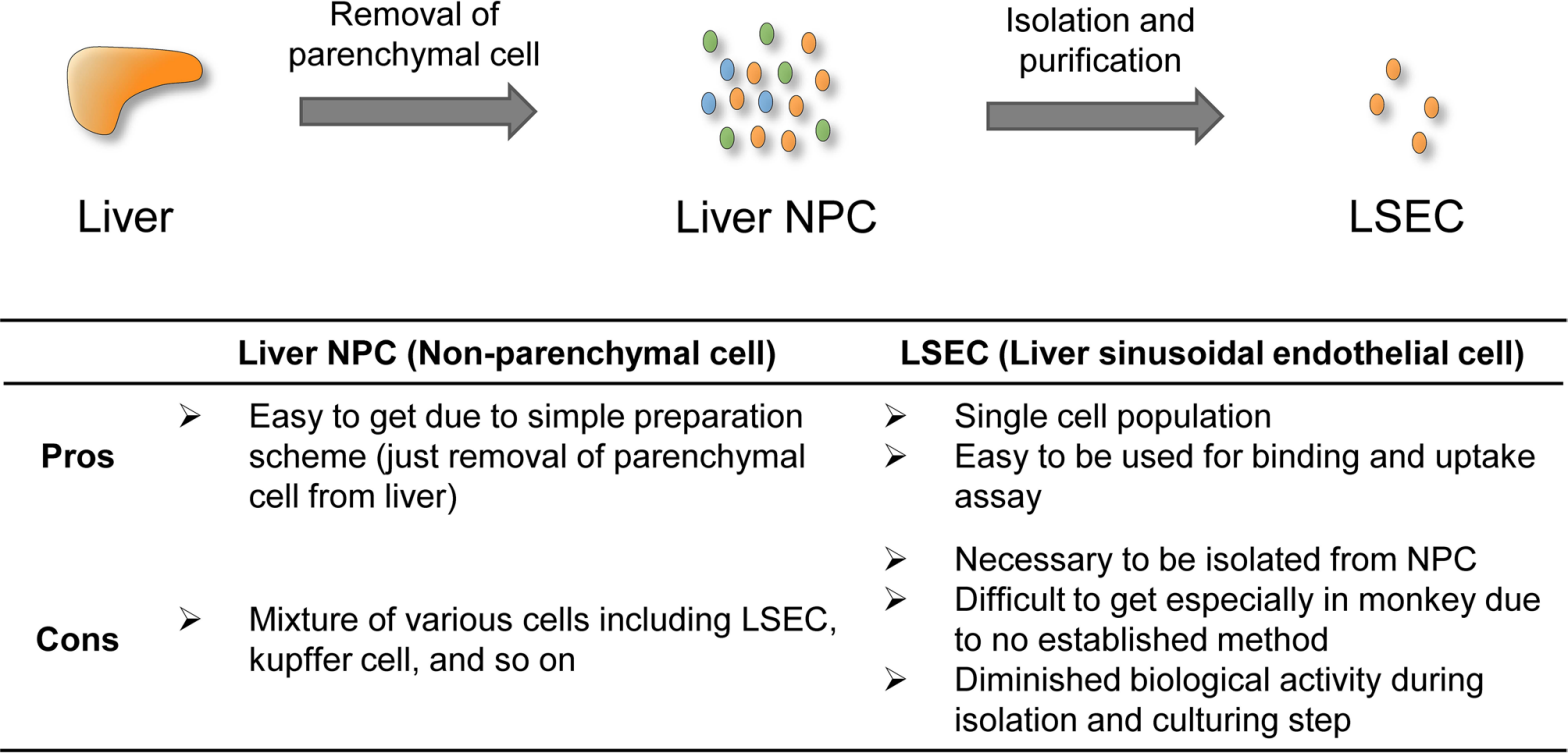
The characteristics of the liver non-parenchymal cells and liver sinusoidal endothelial cells. Liver non-parenchymal cells (NPC) are relatively easy to prepare because their isolation involves only removing parenchymal cells from the liver, however there are hurdles associated with their use for in vitro assays due to the fact that this is a mixture of various types of cells. Liver sinusoidal endothelial cells (LSEC) are appropriate for being used in in vitro assays because they are a single cell population, but it is necessary to be purified from NPC.

However, it is difficult to use in vitro LSEC, especially in monkeys and humans, for binding and uptake assays of an antibody (Fig. 1) since, especially for monkeys and humans, preparing LSEC involves multiple steps including isolation and purification^4,13,14^. Also, during the isolation and culturing processes, the phenotype and biological activity are lost when they are cultured in the absence of other types of feeding cells^15^. Thus, a method for determining the binding and uptake of an antibody by LSEC is an important prerequisite for understanding and/or predicting the elimination process of an antibody.

In this study, we propose an alternative method using NPC (Fig. 1). Large quantities of NPC can be readily obtained in the process of purifying hepatocytes from liver, but this fraction is usually discarded. Since they are composed of a mixture of multiple cell populations, it is generally thought that measuring the binding and uptake of the antibody by LSEC is difficult. To solve this problem, flow cytometry (FCM) was utilized to discriminate between the uptake of the antibodies by the small fraction of LSEC from that by the overall NPC population in the liver. Although there was a problem associated with the quantification in FCM, the fluorochrome-conjugated calibration beads permitted the cellular bound and/or internalized molecules to be quantified^16,17^.

Here, we report on the development of an FCM-based in vitro assay for evaluating the binding and uptake of an antibody against FcγRIIB in LSEC using primary liver NPC from mice and monkeys. In the case of mice, an anti-mouse FcγRIIB antibody (clone 2.4G2)^18,19^ was used. Before the development of the cell-based assay, the in vivo PK profile of this antibody was obtained in wildtype (WT) and FcγRIIB knockout (KO) mice. After the purification of hepatocytes from liver, a suspension of NPC was incubated with an Alexa Fluor 647-labeled anti-FcγRIIB antibody, and the FcγRIIB-expressing cell population was identified by staining with VioBlue-labeled anti-CD45 and FITC-labeled anti-CD146 antibodies, as a mouse LSEC marker^20^. The binding and uptake of the anti-mouse FcγRIIB antibody in the FcγRIIB-expressing cells was then measured to estimate the target-dependent binding (binding affinity (KD) and maximum binding (B_max_)) which are indispensable for accurately estimating the receptor occupancy and the receptor expression level, as well as uptake (Michaelis–Menten constant (K_m_), and maximum uptake velocity (V_max_)) which are essential for evaluating the cellular internalization of the antibody. The obtained in vitro parameters related to uptake were compared to that obtained by the fitting of the in vivo PK profile in WT mice and FcγRIIB KO mice^18^. This scheme was also extended to the corresponding cells from monkeys. The concentration-dependent binding and uptake of the anti-FcγRIIB antibody (clone 046) was determined in monkey NPC. Since no marker for the monkey has been reported, highly homologous markers for human LSEC, i.e. CD31 and CD45, were used to identify the FcγRIIB-expressing cell population^21,22^.

## Results

### The PK profiles of an anti-FcγRIIB antibody in WT and FcγRIIB KO mice

**Figure 2:**
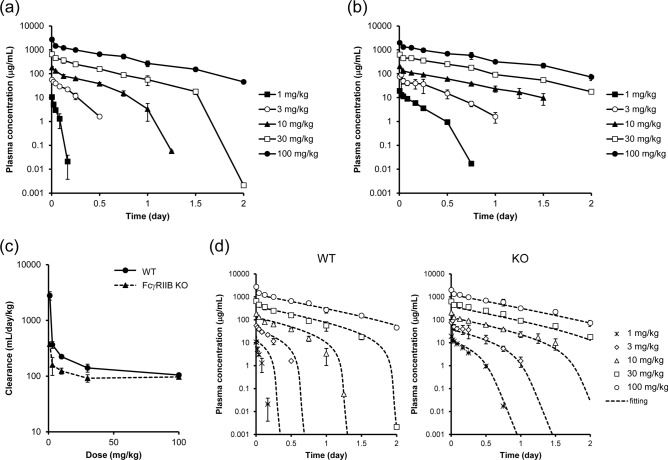
In vivo mouse PK profiles of anti-mouse FcγRIIB antibody. (**a**) Plasma concentration–time profile in wildtype (WT) mice. (**b**) Plasma concentration–time profile in FcγRIIB knockout (KO) mouse. The antibody was intravenously administered at doses of 1, 3, 10, 30, and 100 mg/kg, and plasma was collected time-continuously. The plasma concentrations of the antibody were quantified by ELISA. (**c**) The relationship between clearance and injected dose. (**d**) Simultaneous fitting analysis of WT and KO mice data by using 2-compartment model with Michaelis–Menten equation. Each data point represents the mean ± SD (n = 3).

To characterize the PK profiles of the anti-mouse FcγRIIB antibody (clone 2.4G2), it was intravenously administered to WT and FcγRIIB KO mice in a range of doses from 1 to 100 mg/kg. The plasma concentration–time profiles for the antibody are shown in Fig. 2a,b. In WT mice, non-linear elimination was observed with decreasing dose: the disappearance of the antibody was accelerated. Compared to WT mice, KO mice showed a much slower elimination in the doses from 1 to 30 mg/kg, but the elimination curves for a dose of 100 mg/kg were similar for both the WT and KO mice. These results were also supported by the PK parameters, including clearance and half-life (Supplementary Table 1). The KO mice showed an approximately 7.4-fold smaller clearance at a dose of 1 mg/kg and a longer half-life at doses from 1 to 30 mg/kg. Moreover, as shown in Fig. 2c, the clearance for the WT mice decreased dramatically as the dose approached 100 mg/kg, and a similar clearance for a dose of 100 mg/kg was found for both the WT and KO mice. These data collectively indicate that this antibody is mainly eliminated by the FcγRIIB-mediated pathway at a dose under 30 mg/kg and by other FcγRs such as FcγRIII-dependent and a non-specific pathway at a dose of 100 mg/kg. Figure 2d shows the simultaneous fitting analysis of the WT and KO mice data using the 2-compartment model in which the target specific elimination can be described by using the Michaelis–Menten equation (Eqs. 1 and 2). The estimated K_m_ and V_max_ values by the fitting using these models are shown in Table 1. FcγRIIB-related K_m_ (K_m, FcγRIIB-dep._) and V_max_ (V_max,FcγRIIB-dep._) were estimated to be 0.0971 µg/mL and 1837 µg/day/kg, respectively.

**Table 1:** PK parameters of the WT and FcγRIIB KO mice.

Parameter	Unit	Value	CV (%)
V_1_	mL/kg	46.3	25.0
k_10_	1/day	2.28	25.6
k_12_	1/day	51.4	86.0
k_21_	1/day	76.6	26.6
K_m,FcγRIIB-dep._	µg/mL	0.0971	61.0
V_max,FcγRIIB-dep._	µg/day/kg	1837	4.1
K_m,FcγRIIB-indep._	µg/mL	1.06	19.8
V_max,FcγRIIB-indep._	µg/day/kg	1473	8.8

The mean plasma concentration–time profile of the antibody of WT and FcγRIIB KO mice was simultaneously analyzed by the 2-compartment model with the Michaelis–Menten equation, and the target-independent PK parameters (V_1_, k_10_, k_12_, and k_21_), and the parameters related to FcγRIIB-mediated elimination (K_m,FcγRIIB-dep._ and V_max,FcγRIIB-dep._), and the other FcγRs and unspecific non-linear elimination (K_m,FcγRIIB-indep._ and V_max,FcγRIIB-indep._) were estimated.

### In vitro cellular binding and uptake assay using mouse NPC

**Figure 3:**
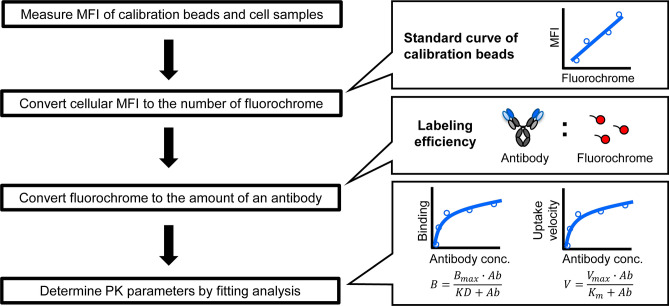
Schematic chart for determining PK parameters in in vitro cell-based assay using flow cytometery. The diagram shows the research flow for determining the amount of the cellular bound and internalized antibody by flow cytometer, and for estimating the PK parameters. MFI, mean fluorescence intensity.

To quantitatively determine the PK parameters for target-specific elimination, an in vitro cell-based assay using mouse NPC was developed, as shown in Fig. 3 and in the Materials and Methods Section. The cell population of interest in NPC was identified by staining the cells with specific cell markers, and the extent of binding and the uptake of the fluorescence-labeled molecules in the target fraction can be quantified by FCM. The mean fluorescence intensity (MFI) of the calibration beads was measured in parallel with the fluorescence of the cellular antibody. Using the calibration curve between the amount of beads and MFI, the MFI that was determined by the fluorescence of the cellular antibody was converted into the actual amount of the antibody. An example of the fluorescence histograms of calibration beads and an antibody, and the scheme for the entire gating are shown in Supplementary Fig. 1. Finally, parameters related to binding (KD and B_max_) which are important parameters for estimating receptor occupancy, and the expression of the receptor and uptake (K_m_ and V_max_) which are needed to evaluate the internalization of an antibody were estimated by a fitting analysis of the titration assay data.

**Figure 4:**
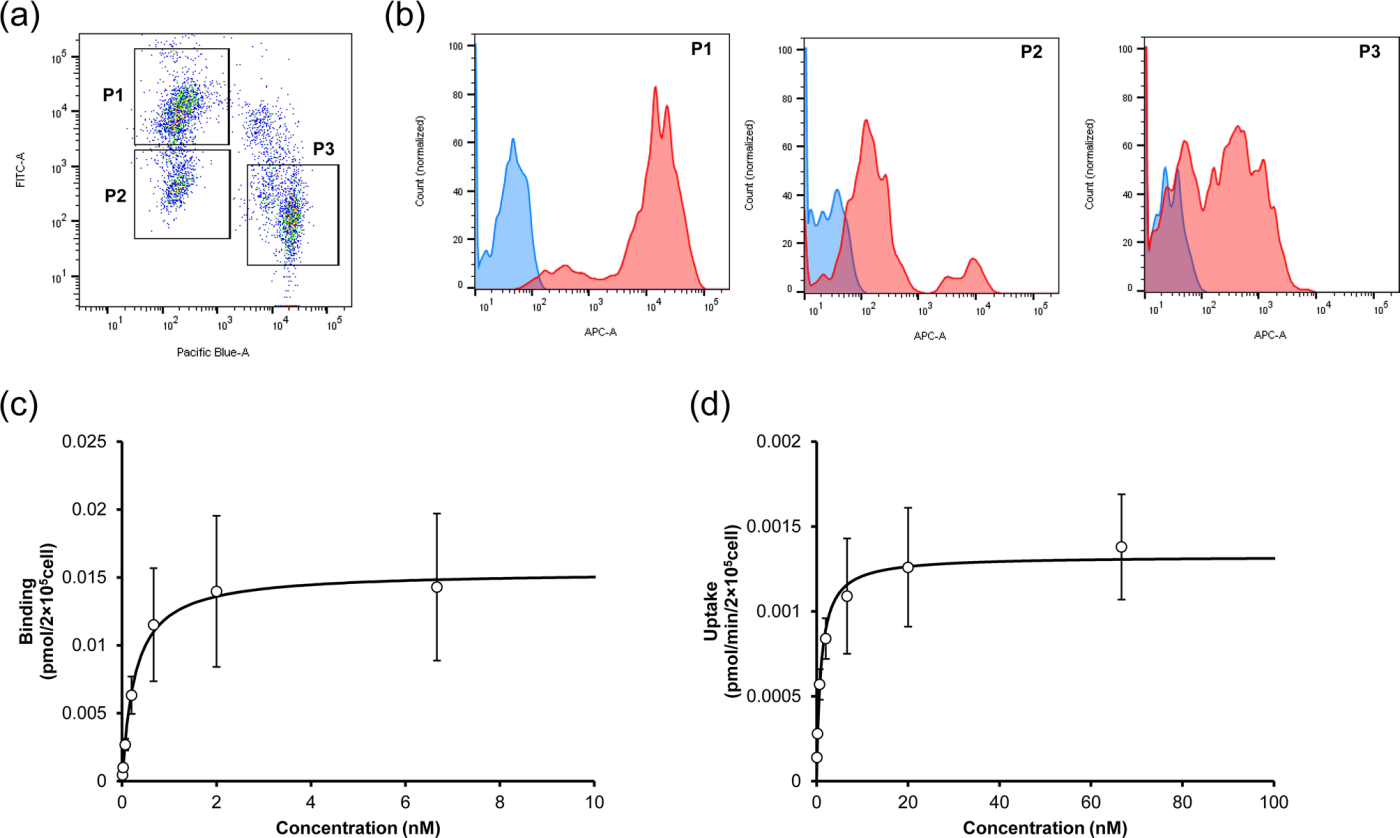
Cellular binding and internalization assay using mouse NPC. (**a**) Cellular dot plot after staining with CD146 and CD45. X axis (Pacific Blue) and Y axis (FITC) shows CD45 and CD146, respectively. CD146 high/CD45 low, CD146 low/CD45 low, and CD146 low/CD45 high population were named as P1, P2, and P3, respectively. (**b**) Histogram of the FcγRIIB expression in each population. X axis (APC) shows the fluorescence of Alexa Fluor 647. Red shows staining with anti- FcγRIIB antibody and blue shows unstaining. (**c**) Concentration-dependent binding of the antibody in P2. The antibody was incubated at 4 °C for 60 min, and fluorescence was detected by FCM. The data were fitted with the receptor-ligand binding equation. (**d**) Concentration-dependent internalization rate of the antibody in P2. The antibody was incubated at 37 °C for 5 min. The data were fitted with the Michaelis–Menten equation. Each point shows the mean ± SD (n = 3).

As shown in Fig. 4a, three cell populations, referred to as P1, P2, and P3, were identified in mouse NPC after the FCM analysis of the cells that had been stained with the CD146 and the CD45 marker, and FcγRIIB expression was then analyzed in all fractions (Fig. 4b). As a result, the P1 (CD146 high/CD45 low) fraction expressed FcγRIIB the highest, while a lower level of expression was found in P2 (CD146 low/CD45 low) and P3 (CD146 low/CD45 high). These data suggest that P1 is the main target cell population expressing FcγRIIB.

To determine the PK parameters for cellular binding and uptake, after being incubated at 4 °C for 60 min, the cell-bound antibody was measured, and the concentration-dependent binding amount was plotted in Fig. 4c. These plots were fitted with the receptor-ligand binding equation (Eq. 3), and KD and B_max_ were estimated as shown in Table 2. Cellular internalization was measured in parallel after incubation at 37 °C for 5 min, and the uptake velocity were plotted against the concentration of the antibody (Fig. 4d). The uptake increased in a concentration-dependent manner, and reached a plateau at a concentration of approximately 20 nM. These plots were analyzed by the Michaelis–Menten equation (Eq. 4). The estimated K_m_ and V_max_ were determined to be 0.969 nM and 0.00133 pmol/min/2 × 10^5^cells, respectively (Table 2).

**Table 2:** PK parameters for the FcγRIIB-mediated binding and uptake in mouse NPC.

Parameter	Unit	Value	CV (%)
KD	nM	0.268	8.7
B_max_	pmol/2 × 10^5^cell	0.0154	2.1
K_m_	nM	0.969	13.3
V_max_	pmol/min/2 × 10^5^cell	0.00133	2.8

The concentration-dependent binding and uptake profiles in mouse NPC were analyzed by the receptor-ligand binding equation and Michaelis–Menten equation, respectively.

### In vitro cellular binding and uptake assay using monkey NPC

**Figure 5:**
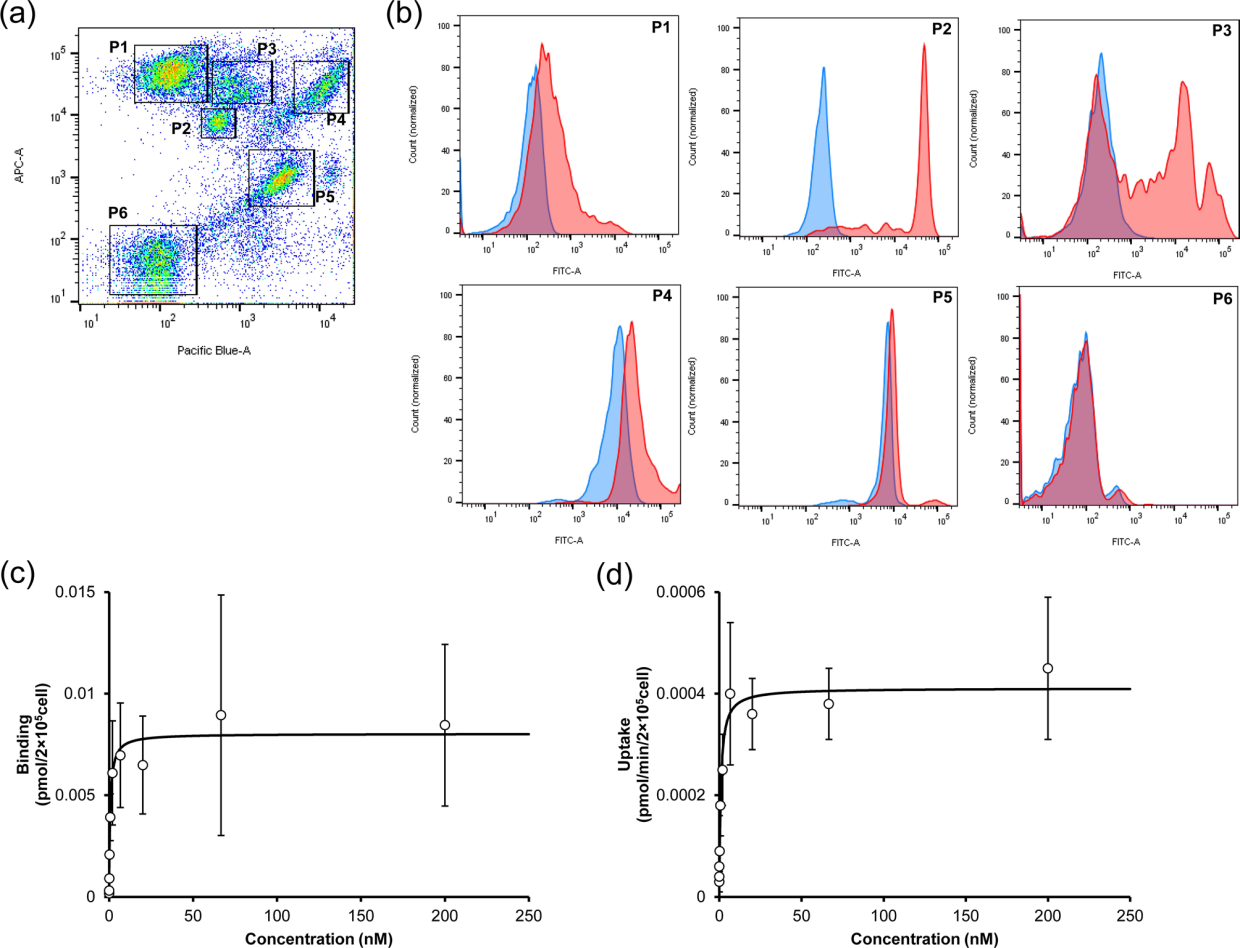
Cellular binding and internalization assay using monkey NPC. (**a**) Cellular dot plot after staining with CD31 and CD45. X axis (Pacific Blue) and Y axis (APC) shows CD31 and CD45, respectively. CD31 low/CD45 high, CD31 mid-low/CD45 mid-high, CD31 mid-low/CD45 high, CD31 high/CD45 high, CD31 mid-high/CD45 mid-low, and CD31 low/CD45 low population were named as P1, P2, P3, P4, P5, and P6, respectively. (**b**) Histogram of the FcγRIIB expression in each population. X axis (FITC) shows the fluorescence of Alexa Fluor 488. Red shows staining with anti- FcγRIIB antibody and blue shows unstaining. (**c**) Concentration-dependent binding of the antibody in P2 cells. The antibody was incubated at 4 °C for 60 min, and fluorescence was detected by FCM. The data were fitted with the receptor-ligand binding equation. (**d**) Concentration-dependent internalization rate of the antibody in P2 cells. The antibody was incubated at 37 °C for 10 min. The data were fitted with the Michaelis–Menten equation. Each point shows the mean ± SD (n = 3).

To extend this FCM-based method to estimating the K_m_ and V_max_ in monkey cells, an in vitro assay using monkey NPC was performed. As shown in Fig. 5a, six cell clusters (P1–P6) were identified by staining with the CD31 and CD45 marker and the expression of FcγRIIB was analyzed for each population (Fig. 5b). As a result, P2 (CD31 mid-low/CD45 mid-high) showed the highest expression of FcγRIIB. A positive peak of FcγRIIB was also found in a small fraction (around 50%) of cells in P3 (CD31 mid-low/CD45 high). P1 (CD31 low/CD45 high), P4 (CD31 high/CD45 high), P5 (CD31 mid-high/CD45 mid-low), and P6 (CD31 low/CD45 low) showed either a low or no expression. These findings suggest that P2 is a target cell for an anti-FcγRIIB antibody.

To quantitatively estimate the binding and uptake parameters for the FcγRIIB-mediated elimination in monkey cells, a titration assay of anti-monkey FcγRIIB antibody (clone 046) was performed by using the P2 fraction. A typical example of the detected fluorescence histograms and the entire gating scheme is shown in the Supplementary Fig. 2. Concentration-dependent binding (4 °C, 60 min) and uptake (37 °C, 10 min) was observed as shown in Fig. 5c,d. These plots were analyzed with the receptor-ligand binding equation (Eq. 3) for binding, and the Michaelis–Menten equation (Eq. 4) for uptake. From these fitting curves, the binding and uptake-related parameters (KD, B_max_, K_m_, and V_max_) in monkey NPC were estimated and the results are shown in Table 3.

**Table 3:** PK parameters for the FcγRIIB-mediated binding and uptake in monkey NPC.

Parameter	Unit	Value	CV (%)
KD	nM	0.670	21.3
B_max_	pmol/2 × 10^5^cell	0.00803	3.8
K_m_	nM	0.891	24.2
V_max_	pmol/min/2 × 10^5^cell	0.000411	4.4

The concentration-dependent binding and uptake profiles in monkey NPC were analyzed by the receptor-ligand binding equation and Michaelis–Menten equation, respectively.

## Discussion

The purpose of this study was to establish a cell-based assay that allows binding and uptake of an antibody to be evaluated on FcγRIIB-expressing cells using liver NPC. The FcγRIIB-expressing population was identified in mice by staining with CD146 and CD45. The amount of binding and uptake in the target population was quantified and PK parameters (KD, B_max_, K_m_, and V_max_) were determined. Similar to mice, monkey NPC were stained with CD31 and CD45 to identify the FcγRIIB-expressing cells, and a concentration-dependent binding and the uptake of an antibody in the target fraction was confirmed, thus allowing the PK parameters to be successfully determined.

We demonstrated that PK parameters could be obtained by a mouse NPC binding and uptake assay using FCM, and that the obtained values were consistent with the in vivo values. We used an anti-mouse FcγRIIB antibody (2.4G2) as a model antibody for validation in mice, although the 2.4G2 antibody has been reported to bind FcγRIII in addition to FcγRIIB, and thus it is possible that the expression of FcγRIII could affect the in vivo PK and in vitro cellular binding/uptake of the antibody in this research^18,19,23^. We first characterized this antibody in vivo. As shown in Fig. 2a,b, a much faster elimination was observed in the WT mice compared to the KO mice in a dose range from 1 to 30 mg/kg, and the KO mice showed an up to 7.4 times smaller clearance compared to the WT mice (Fig. 2c and Supplementary Table 1). These results indicate that the FcγRIIB-mediated pathway is the major contributor to the elimination of this antibody. In addition, a PK analysis showed that the K_m,FcγRIIB-dep._ was 10.9-fold smaller than the K_m,FcγRIIB-indep._, and that the V_max,FcγRIIB-dep._ was 1.2-fold larger than V_max,FcγRIIB-indep._ (Fig. 2d and Table 1), which suggests that the antibody has a higher affinity and a larger capacity against FcγRIIB compared to those against other FcγRs such as FcγRI, FcγRIII, and/or FcγRIV. On the other hand, at a dosage of 100 mg/kg, a similar PK profile was observed both in WT mice and KO mice (Fig. 2a,b). Additionally, Fc gamma chain knockout (FcGC KO) mice at a dose of 100 mg/kg showed comparable PK profiles and clearances to those of WT and FcγRIIB KO mice (Supplementary Fig. 3 and Supplementary Table 1), which suggests that FcγRIIB-mediated elimination is saturated even in FcGC KO mice in this dosage. These findings suggest that this antibody is eliminated mainly via FcγRIIB, although there is the minor contribution of other FcγRs.

Developing a cell-based assay for evaluating the PK of an antibody has become an active area of research. For example, an FcRn-mediated recycling assay and an FcγRIIB-mediated uptake assay using overexpressing cell lines has been reported^18,24,25^. However, there is currently no method available for estimating binding and uptake-related parameters that are useful for the prediction of PK using primary cells with fluorescence detection which is easy to handle. As shown in Fig. 3, fluorescence detection of calibration beads by FCM in parallel with detecting the cellular fluorescence of an antibody enabled us to determine the cellular binding and the amount of the antibody that is taken up, and to estimate PK parameters quantitatively in vitro.

In Fig. 4, cellular binding and uptake in FcγRIIB-expressing cells were evaluated by FCM after staining with CD146 and CD45, and the KD, B_max_, K_m_, and V_max_ were determined (Table 2). P1 cells showed the highest expression of FcγRIIB, which suggests that this fraction is the target site (i.e. LSEC) for this antibody, although this result might include the expression of other FcγRs such as FcγRIII due to the binding properties of the 2.4G2 antibody. The KD was estimated to be 0.268 nM by a binding assay, which is similar to that for the Biacore assay^23^. Therefore, an FCM-based assay using NPC is appropriate to measure binding affinity, and additionally, the level of expression can be determined from the B_max_ value, which cannot be determined by a Biacore assay. Figure 4d shows the uptake profile, and the K_m_ and V_max_ values were determined (Table 2). To compare the in vitro parameters to the in vivo parameters, the in vitro K_m_ and V_max_ values were adjusted for the molecular weight, the number of cells, and the body weight of the mice. As a result, only 1.5-fold differences in the K_m_ were found between the in vitro (0.145 µg/mL) and in vivo (0.0971 µg/mL) values. This, therefore, suggests that this assay will be useful for estimating the K_m_. On the other hand, 3.4-fold differences between in vitro (544 µg/day/kg) and in vivo (1837 µg/day/kg) were found for V_max_. This is because cellular activity might have decreased during the isolation steps, indicating that a method with less cell damage is needed. It has been reported that the cellular activity of primary liver cells decreases in a time dependent manner^26,27^. Furthermore, the expression of FcγRIIB (and other FcγRs such as FcγRI, FcγRIII, and/or FcγRIV) was low even in P2 and P3 cells, which may contribute to the disappearance of the antibody in vivo, although the expression level was much lower than P1. The combined values for V_max_ in P1 to P3 cells might contribute to a more accurate estimation of V_max_.

Similar to the case of the mouse, an NPC assay using monkey NPC was performed to determine whether the methodology would be applicable for determining PK parameters in monkeys. Although a human LSEC marker is known, a monkey version is not. Therefore, a human LSEC marker (CD31 and CD45) was used to identify FcγRIIB-expressing cells. As shown in Fig. 5a,b, only P2 expressed high levels of FcγRIIB, while P1, P4, P5, and P6 did not. P3 showed two peaks, but in any case, the expression level was weak. Although other staining methods should be investigated to clarify the details associated with the separation of each fractions in the future, the above findings clearly indicate that the P2 fraction is the target cell. Thus, concentration-dependent antibody binding and uptake was found in P2 fraction, thus allowing the binding and uptake parameters to be determined (Fig. 5c,d, and Table 3). This is the first example of the determination of antibody PK parameters using primary monkey NPC.

However, there are some limitations for this assay that uses NPC. In this study, only an antibody against FcγRIIB was evaluated. In the mice study, the 2.4G2 antibody was used as a model antibody. Since the in vitro uptake and in vivo PK profile of the 2.4G2 antibody may be affected by binding to FcγRIII, as well as FcγRIIB, it would be advisable to validate the in vitro and in vivo studies by using more FcγRIIb-specific antibodies like Ly17.2 or Ly17.1, or AT130 to distinguish the contributions of FcγRIIB and FcγRIII to the overall clearance of the antibody detail^23^. Moreover, it is the specific binder to FcγRIIB as the direct target of the antibody, that is recognized by its variable Fab regions. In addition, the apparent affinity of the bivalent antibody might be different from that for FcγRs-mediated monovalent binding via Fc domain. Thus, this may not reflect the antibody clearance mechanisms via the Fc domain. Therefore, additional studies using antibodies that do not recognize mouse antigens, and are dominantly taken up by the FcγRs via Fc domain of the antibody would be needed to extrapolate our method to the conventional antibody and/or immune complex. Furthermore, applications for antibodies against other target proteins including non-target binding antibodies should also be investigated in the future. Since it is generally thought that several receptors are expressed in NPC, this assay has the potential for being used to evaluate the PK parameters of other antibodies as well. In addition, to reveal the predictability of use in monkeys, more detailed studies including in vivo and in vitro experiments will be needed to validate the PK parameters obtained by NPC assay.

On the other hand, our assay would be useful for predicting the PK values for monkeys and humans by performing in vitro assays using monkey and human NPC. This is because the methodology and tools for predicting PK, especially human PK, based on not only in vivo but also in vitro assays are not well-established. This methodology is expected to be extended to determining human PK parameters using human NPC in the future. The monkey assay can also contribute to the 3Rs for reducing the need for experimental monkeys. In addition, this NPC assay can be applied not only to evaluating the PK of an antibody, but also that of other substances. Since LSEC is involved in the uptake of oligonucleotides^7,8^, and viruses^11^, these binding and uptake assays using NPC could be helpful and could also be used for pharmacological and toxicological evaluation.

In conclusion, we report on the development of a novel FCM-based cellular assay using mouse and monkey NPC for evaluating the binding and uptake of an antibody, and determining PK parameters. In mice, it was possible to evaluate the concentration-dependent binding and uptake in FcγRIIB-expressing cells by staining with CD146 and CD45, and it was confirmed that in vitro PK parameters were correlated with the in vivo values. In addition, this scheme was extended to monkeys, where staining with CD31 and CD45 led to the identification of the FcγRIIB-expressing population, and to reveal concentration-dependent binding and uptake of the antibody. This assay is expected to be used for determining PK parameters and predicting PK profiles for an antibody in the area of drug discovery.

## Materials and methods

### Reagents

The following materials were purchased from commercial sources: Clear Immuno 384-Well Plate (Thermo Scientific, 8755), an anti-human IgG antibody (Bethyl Laboratories, A80-319A), Streptavidin-PolyHRP80 (Stereospecific Detection Technologies, SP80D50), Bovine Serum Albumin (BSA) for ELISA (Sigma-Aldrich, A7030), TMB Substrate (Surmodics, TMBS-1000–01), Carbonate-Bicarbonate Buffer capsule (Sigma-Aldrich, C3041), 96-well cell culture plate (Costar, 3799), BSA for cell assay (Sigma-Aldrich, A9418), Fetal bovine serum (FBS) (Sciencell, 0500), anti-mouse CD45 antibody-VioBlue (Miltenyi, 130-110-664), anti-mouse CD146 antibody-FITC (Miltenyi, 130-102-230), anti-human CD31 antibody-Pacific Blue (BioLegend, 303114), anti-non human primate CD45 antibody-APC (Miltenyi, 130-091-900), anti-CD32B (FcγRIIB) antibody (Sino Biological, 90014-R046), Liver Perfusion Medium (Gibco, 17701-038), Endothelial cell medium (Sciencell, 1001), Hepatocyte Culture Medium (Lonza, CC-3198), Alexa Fluor 488 Labeling Kit (Invitrogen, A20181), Alexa Fluor 647 Labeling Kit (Invitrogen, A20173). Other reagents were purchased from local commercial sources.

### Animals

WT (C57BL/6NTac), FcγRIIB KO (Fcgr2b), and FcGC KO (Fcer1g) mice (6 weeks, male) were purchased from the Taconic Biosciences, Inc.

### Animal experiments

The animal studies were carried out in compliance with the ARRIVE guidelines (https://arriveguidelines.org/). All procedures in this study were reviewed and approved by the Institutional Animal Care and Use Committee (IACUC) in Chugai Pharmaceutical Co., Ltd. The animal experiments were performed in accordance with the Guidelines for the Care and Use of Laboratory Animals at Chugai Pharmaceutical Co., Ltd, which is accredited by the Association for Assessment and Accreditation of Laboratory Animal Care (AAALAC) International. The animal experiments in this study were performed in Sekisui Medical Co., Ltd.

### PK study of the anti-mouse FcγRIIB antibody in mice

These experiments were carried out with reference to a previous report^18^. In brief, the PK of the anti-mouse FcγRIIB antibody in plasma was evaluated by administering the antibody at doses of 1, 3, 10, 30, and 100 mg/kg to WT, FcγRIIB KO, and FcGC KO mice via the tail vein. The administered volume was 10 mL/kg. At 5 min, 30 min, 1 h, 2 h, 3 h, 4 h, 6 h, 12 h, 18 h, 24 h, 30 h, 36 h, and 48 h after the injection, blood was collected from the cervical vein without anesthesia and mixed with heparin sodium. Plasma was obtained by centrifugation of the blood (1800 × *g*, 4 °C, 15 min).

### Measurement of the antibody in plasma samples by ELISA

The antibody concentrations in plasma samples were measured in a sandwich ELISA. Anti-human IgG antibody (Bethyl Laboratories, A80-319A) was applied to a Clear Immuno 384-Well Plate (Thermo Scientific, 8755) at the concentration of 1 µg/mL in carbonate-bicarbonate buffer (Sigma-Aldrich, C3041). After incubation for overnight at 4 °C, the solution was removed and washed with 1% BSA-Phosphate buffered saline containing Tween 80 (PBST), following blocking with 1% BSA-PBST. Diluted plasma samples were then incubated with a biotin-labeled anti-human IgG Fc antibody (In house preparation). Poly HRP-labeled streptavidin (Stereospecific Detection Technologies, SP80D50) was added, and finally TMB substrate (Surmodics, TMBS-1000-01) was applied. After stopping the reaction by adding 0.5 M sulfuric acid, absorbance was detected by spectrophotometer (BMG Labtech, German).

### PK analysis of mice PK data

Phoenix 64 WinNonlin 8.2 (ver. 8.2.0.4383) was used to analyze the mouse PK data. To reveal the half-life, the concentration at time zero (C_0_), the area under the curve (AUC_0-inf_), clearance, and volume of distribution (V_d_), non-compartment model analysis was done. Simultaneous fitting analysis was also performed for WT and FcγRIIB KO mice by using the 2-compartment model with Michaelis–Menten equation, where FcγRIIB-dependent and FcγRIIB-independent elimination were included as shown in Eq. (1) and (2).

1 $${V}_{1}\cdot \frac{dAb}{dt}=-{k}_{10}\cdot Ab\cdot {V}_{1}+{k}_{21}\cdot {X}_{Ab,peripheral}-{k}_{12}\cdot Ab\cdot {V}_{1}-\frac{{V}_{max,Fc\gamma RIIB-dep.}\cdot Ab}{{K}_{m,Fc\gamma RIIB-dep.}+Ab}-\frac{{V}_{max,Fc\gamma RIIB-indep.}\cdot Ab}{{K}_{m,Fc\gamma RIIB-indep.}+Ab}$$

2 $$\frac{{X}_{Ab,peripheral}}{dt}=-{k}_{21}\cdot {X}_{Ab,peripheral}+{k}_{12}\cdot Ab\cdot {V}_{1}$$

here V_1_ and k_10_ indicates the central compartment volume and elimination rate constant, respectively. k_12_ and k_21_ indicate distribution rate constants. K_m,FcγRIIB-dep._ and V_max,FcγRIIB-dep._ mean the Michaelis–Menten constant and maximum velocity on Fc_γ_RIIB-dependent non-linear pathway. K_m,FcγRIIB-indep._ and V_max,FcγRIIB-indep._ denote those on a target-independent non-linear pathway.

### Labeling of the antibody with Alexa Fluor 488 and 647

The antibody was labeled with Alexa Fluor 488 and 647 using an Antibody Labeling Kit (Invitrogen, A20181 and A20173). The operation was basically performed according to the attached document. Briefly, the antibody was incubated with an Alexa Fluor dye and the resulting reaction solution was purified by a Zeba Spin Column (Thermo Scientific, 89883) to remove the free dye. The concentration of the antibody and labeling efficiency was determined by a NanoDrop Spectrophotometer (Thermo Scientific, United States).

### In vitro binding and internalization assay using mouse NPC

Mouse NPC were prepared as described in a previous report^4,13,14^. Briefly, mouse livers were perfused with Liver Perfusion Medium (Gibco, 17701-038) to remove blood, and then perfused with a Collagenase solution (Wako, 034-22363) to digest the tissue. The cell suspension was centrifuged (50 × *g*, 4 °C, 3 min) three times to remove parenchymal cells. The supernatant was finally centrifuged (300 × *g*, 4 °C, 5 min), and the pellet was suspended in Endothelial cell medium (Sciencell, 1001).

To detect the cellular binding and uptake of the antibody, the Alexa Fluor 647-labeled anti-mouse FcγRIIB antibody was incubated with the mouse NPC for binding (4 °C, 60 min) and uptake (37 °C, 5 min). After the reaction, the cells were washed and stained with an anti-mouse CD146 antibody-FITC (Miltenyi, 130-102-230) and anti-mouse CD45 antibody-VioBlue (Miltenyi, 130-110-664). Finally, the fluorescence intensity of the samples was detected by BD FACSCanto II (Becton, Dickinson and Company, United States). In parallel with detecting the cellular signal, the fluorescence of the calibration beads (Bangs Laboratories, 647) was also measured. Cellular fluorescence was converted to the amount of antibody by using the calibration curve of standard beads and the labeling efficiency.

### In vitro binding and internalization assay using monkey NPC

Monkey NPC was purchased from Ina Research, Inc. Briefly, livers were perfused with Hanks-HEPES buffer and a Collagenase solution. The cell suspension was centrifuged (50 × *g*, 4 °C, 1 min) twice, and finally centrifuged (1000 × *g*, 4 °C, 5 min) to give NPC. The NPC cells were then purified by density-gradient centrifuge, and suspended in Hepatocyte culture medium (Lonza, CC-3198).

To detect the cellular binding and uptake of the antibody in monkey NPC, the Alexa Fluor 488-labeled anti-monkey FcγRIIB antibody (Sino Biological, 90014-R046) was incubated with the monkey NPC to allow binding (4 °C, 60 min) and uptake (37 °C, 10 min) to occur. After the reaction, cells were washed and stained with anti-human CD31 antibody-Pacific Blue (BioLegend, 303114) and anti-non human primate CD45 antibody-APC (Miltenyi, 130-091-900). Finally, the fluorescence intensity of the samples was detected by BD FACSCanto II (Becton, Dickinson and Company, United States). In parallel with detecting cellular signal, the fluorescence of the calibration beads (Bangs Laboratories, 488) was detected.

### Fitting analysis of in vitro NPC binding and uptake data

Phoenix 64 WinNonlin 8.2 (ver. 8.2.0.4383) was used to analyze the in vitro data. To reveal the KD and B_max_, the concentration-dependent binding data was analyzed by the receptor-ligand binding Eq. (3).

3 $${Ab}_{bound}=\frac{{B}_{max}\cdot Ab}{KD+Ab}$$

here KD and B_max_ indicate the binding affinity and the maximum binding amount, respectively.

To reveal the K_m_ and V_max_, the concentration-dependent uptake data was analyzed by the Michaelis–Menten Eq. (4).

4 $$V=\frac{{V}_{max}\cdot Ab}{{K}_{m}+Ab}$$

here K_m_ and V_max_ indicate the Michaelis–Menten constant and the maximum elimination rate, respectively. To convert the in vitro V_max_ (pmol/min/2 × 10^5^ cells) to the in vivo value, in vitro V_max_ was compensated by the number of cells (7.6 × 10^6^ cells, including LSEC and liver KC which express FcγRIIB mainly in the mouse)^28^ and the body weight of the mice.

## Supplementary Information

Supplementary Information.

## Data Availability

The datasets generated in this study are available from the corresponding author upon reasonable request.
