# Supplementary material for: A cell based assay for evaluating binding and uptake of an antibody using hepatic nonparenchymal cells
Source: Sci Rep. 2021 Apr 16;11:8383. doi: 10.1038/s41598-021-87912-6 (PMC8052349; doi:10.1038/s41598-021-87912-6)
Supplement: Supplementary file 1 — Supplementary Information. [file 41598_2021_87912_MOESM1_ESM.docx]

**Supplementary**

**A cell based assay for evaluating binding and uptake of an antibody using hepatic nonparenchymal cells**

Yuki Noguchi^1,2^, Kazuhisa Ozeki^1*^, Hiroaki Takesue^1^, Hidetaka Akita^2^

**Supplementary Fig. 1 The example results of the flowcytometry histogram of the calibration beads and binding/uptake of the antibody in mouse NPC**

(a) The histogram of the Alexa Fluor 647 calibration beads. Each color shows the different number of fluorochrome-modified beads. (b) The histogram of the binding of the antibody in mouse NPC. (c) The histogram of the uptake of the antibody in mouse NPC. (d) The entire gating scheme for mouse NPC by FCM and the dot plots of CD45 and CD146 in stained/unstained control.


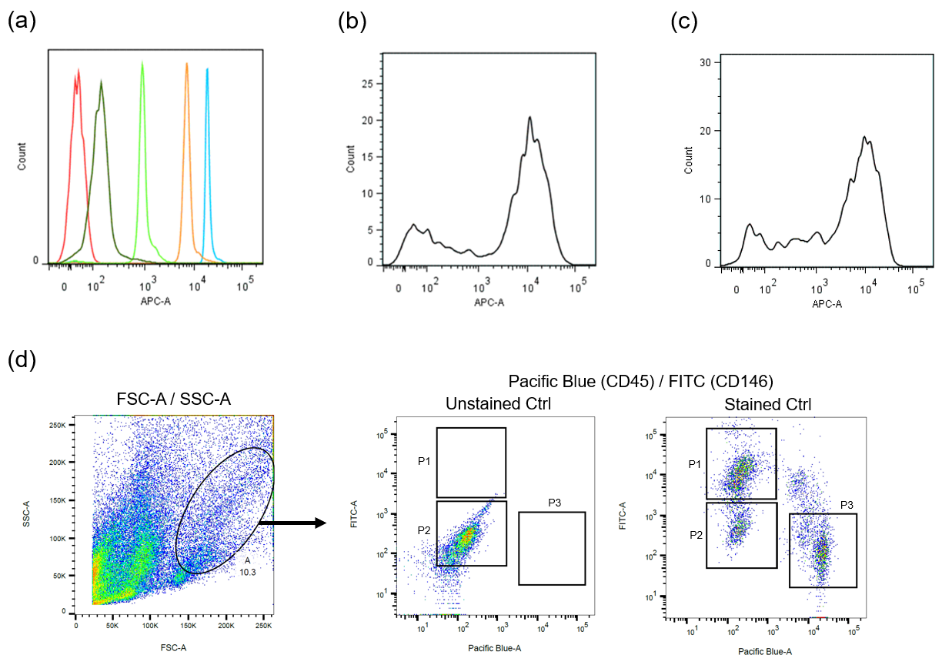


**Supplementary Fig. 2 The example results of the flowcytometry histogram of the calibration beads and binding/uptake of the antibody in monkey NPC**

(a) The histogram of the Alexa Fluor 488 calibration beads. Each color shows the different number of fluorochrome-modified beads. (b) The histogram of the binding of the antibody in monkey NPC. (c) The histogram of the uptake of the antibody in monkey NPC. (d) The entire gating scheme for monkey NPC by FCM and the dot plots of CD31 and CD45 in stained/unstained control.


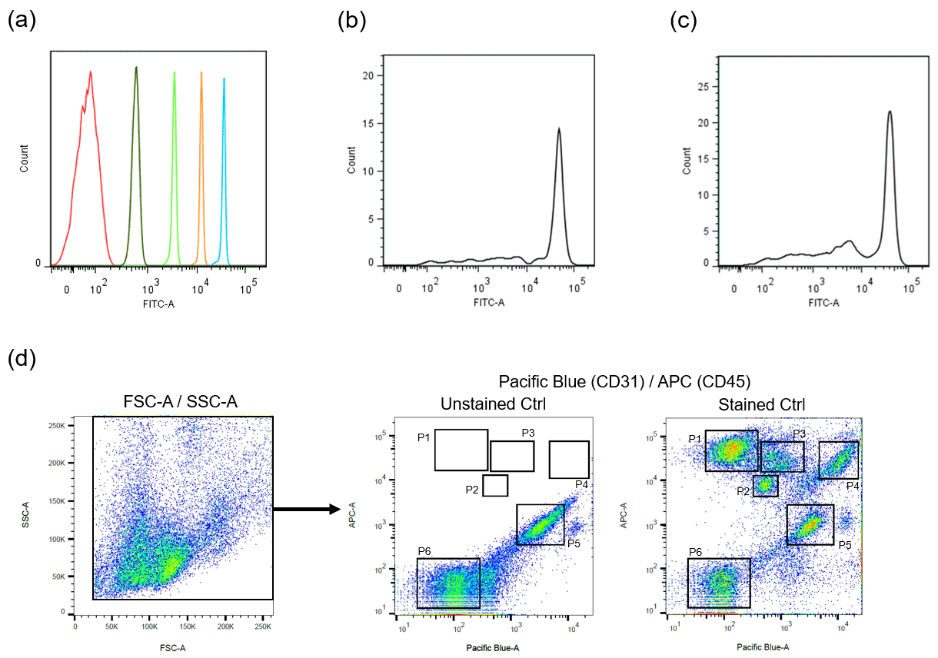


**Supplementary Fig. 3 The PK profile of the 2.4G2 antibody in Fc gamma chain KO mice**

The 2.4G2 antibody was intravenously administered to Fc gamma chain subunit knockout (FcGC KO) mice in a range of doses from 1 to 100 mg/kg. Plasma concentrations were measured by ELISA.

**Supplementary Table 1 Non-compartmental PK parameters of the antibody in WT, FcγRIIB KO, and FcGC KO mice**

| **Mouse** | **Dose** | **Half-life** | **C_0_** | **AUC_0-inf_** | **Clearance** | **V_d_** |
| --- | --- | --- | --- | --- | --- | --- |
|  | **(mg/kg)** | **(day)** | **(μg/mL)** | **(day*μg/mL)** | **(mL/day/kg)** | **(mL/kg)** |
| **WT** | **1** | 0.02 ± 0.00 | 12 ± 2 | 0.4 ± 0 | 2795 ± 482 | 88 ± 10 |
|  | **3** | 0.08 ± 0.02 | 59 ± 8 | 8 ± 1 | 366 ± 52 | 51 ± 5 |
|  | **10** | 0.05 ± 0.03 | 189 ± 19 | 45 ± 4 | 224 ± 18 | 63 ± 4 |
|  | **30** | 0.25 ± 0.19 | 673 ± 64 | 216 ± 32 | 141 ± 23 | 62 ± 10 |
|  | **100** | 0.36 ± 0.08 | 2914 ± 715 | 957 ± 90 | 105 ± 9 | 60 ± 6 |
| **FcγRIIB KO** | **1** | 0.10 ± 0.04 | 21 ± 2 | 3 ± 0 | 376 ± 11 | 64 ± 3 |
|  | **3** | 0.14 ± 0.02 | 79 ± 15 | 21 ± 9 | 159 ± 56 | 45 ± 16 |
|  | **10** | 0.38 ± 0.10 | 186 ± 49 | 81 ± 11 | 124 ± 15 | 69 ± 5 |
|  | **30** | 0.38 ± 0.01 | 653 ± 80 | 333 ± 48 | 92 ± 14 | 54 ± 8 |
|  | **100** | 0.43 ± 0.06 | 2052 ± 287 | 1032 ± 81 | 97 ± 8 | 66 ± 6 |
| **FcGC KO** | **1** | 0.01 ± 0.00 | 9 ± 1 | 0.1 ± 0 | 7347 ± 248 | 113 ± 10 |
|  | **3** | 0.08 ± 0.05 | 58 ± 10 | 7 ± 0 | 448 ± 9 | 62 ± 19 |
|  | **10** | 0.03 ± 0.00 | 188 ± 38 | 34 ± 2 | 297 ± 17 | 49 ± 4 |
|  | **30** | 0.09 ± 0.06 | 539 ± 149 | 166 ± 45 | 191 ± 60 | 59 ± 17 |
|  | **100** | 0.22 ± 0.06 | 1917 ± 167 | 803 ± 68 | 125 ± 11 | 63 ± 7 |

The PK profiles of the 2.4G2 antibody in WT, FcγRIIB KO, and FcGC KO mice were analyzed with the non-compartment model (moment) analysis. Half-life, the concentration at time zero (C_0_), the area under the curve (AUC_0-inf_), clearance, and volume of distribution (V_d_) were shown. The data represent mean ± SD (n = 3).
